# Bone morphogenetic protein 10 alleviates doxorubicin-induced cardiac injury via signal transducer and activator of transcription 3 signaling pathway

**DOI:** 10.1080/21655979.2022.2048994

**Published:** 2022-03-16

**Authors:** Peng An, Di Fan, Zhen Guo, Fang-Yuan Liu, Chen-Fei Li, Dan Yang, Ming-Yu Wang, Zheng Yang, Qi-Zhu Tang

**Affiliations:** a Department of Cardiology, Renmin Hospital of Wuhan University, Wuhan, RP China; b Hubei Key Laboratory of Metabolic And Chronic Diseases, Wuhan, RP China; c Cardiovascular Research Institute of Wuhan University, Wuhan 430060, RP China

**Keywords:** BMP10, doxorubicin, STAT3, oxidative stress, apoptosis, cardiac injury

## Abstract

Doxorubicin (DOX) has limited antitumor applications owing to its association with life-threatening cardiac injury. Oxidative damage and cardiac apoptosis are crucial in DOX-induced cardiac injury. Bone morphogenetic protein 10 (BMP10) is predominantly distributed in the heart and acts as a cardioprotective factor that preserves cardiac function. However, the role of BMP10 in DOX-induced cardiac injury has not yet been explored. The current study aimed to examine the function and mechanism of action of BMP10 in DOX-induced cardiac injury. An adeno-associated viral system was used for the overexpression or silencing of cardiac-specific BMP10, and subsequently, a single dose of DOX was intraperitoneally injected to induce cardiac injury. Results showed that DOX exposure decreased BMP10 expression in the heart. Cardiac-specific overexpression of BMP10 alleviated the oxidative stress and apoptosis and improved cardiac function. Conversely, cardiac-specific silencing of BMP10 aggravated the redox disorder and apoptosis and worsened the cardiac dysfunction caused by DOX. Exogenous BMP10 supplementation amelioratesd the DOX-induced cardiac contractile dysfunction. Mechanistically, we found that phosphorylation of signal transducer and activator of transcription 3 (STAT3) is reduced in DOX-induced cardiotoxicity, and, BMP10 activated impaired STAT3 via a non-canonical pathway. BMP10 lost its cardioprotective function in cardiomyocyte-specific STAT3 knockout (STAT3-cKO) mice. Based on our findings, we suggested that BMP10 is a potential therapeutic agent against DOX-induced cardiac injury and that the cardioprotective effects of BMP10 are dependent on the activation of STAT3.

## GraphicalAbstract

**Figure:**
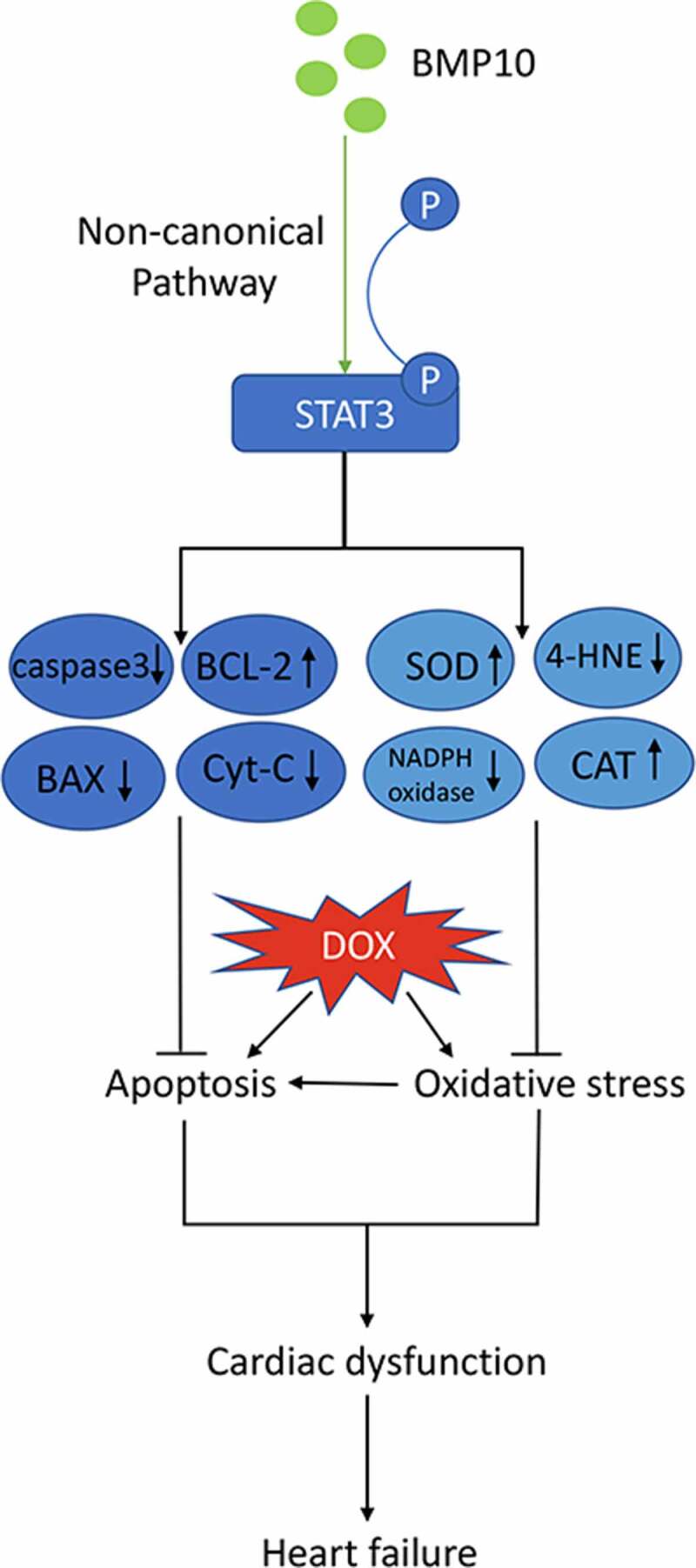

## Introduction

Doxorubicin (DOX) is a broad-spectrum antitumor drug with excellent curative effects on breast cancer and hematological malignancies [1, 2]. Unfortunately, the irreversible and fatal cardiotoxicity of DOX limits its clinical application [3]. DOX is considered to cause cardiomyopathy and systolic cardiac dysfunction [4, 5]. Multiple mechanisms have been confirmed to be involved in DOX cardiotoxicity, including DNA synthesis inhibition, energy metabolism disorders and endoplasmic reticulum stress [^6–8^]. However, recent evidence has revealed myocardial oxidative stress and apoptosis as essential factors in DOX cardiac injury [9]. DOX leads to excessive production of reactive oxygen species (ROS) [10], which damage DNA and destroy cell membrane integrity [11]. In addition, DOX-induced oxidative stress triggers substantial cardiac apoptosis, leading to life-threatening cardiac dysfunction [12]. Despite numerous studies focusing on DOX cardiotoxicity, effective therapies against DOX-induced cardiac injury are not yet available [13]. Therefore, it is important to identify novel therapeutic targets for treating DOX-related cardiac injury.

As a member of the signal transducer and activator of transcription (STAT) family, STAT3 was initially confirmed as a cytoplasmic transcription factor that is activated by extracellular signaling [14]. STAT3 is constitutively expressed in cardiomyocytes and regulates the physiological and pathological cardiac processes [15]. It is considered a cardioprotective factor owing to its activation of antioxidant enzymes and induction of anti-apoptotic proteins [16]. STAT3 activation protects cardiomyocytes from ischemia-reperfusion injury [17]. High expression of STAT3 helps cardiomyocytes in tolerating H_2_O_2_ stimulation, and hence, decreases apoptosis [18]. Recent evidence suggests that STAT3 is involved in mitochondrial energy metabolism through interactions with the electron transport chain [19]. Taken together, the antioxidant defense mechanism and additional cytoprotective pathways of STAT3 play critical roles in cardiomyocyte survival and cardiac function maintenance. STAT3 activation has been shown to alleviate DOX-induced acute cardiac injury [20]. STAT3 acts as a protective signal against DOX-induced cardiomyopathy by inhibiting reduction of cardiac contractile genes and inducing cardiac protective factors [21]. Therefore, STAT3 may be a potential therapeutic target for treating DOX-induced cardiotoxicity, and exploration of an effective and positive STAT3 regulator would be of great significance.

It has been reported that BMP10 significantly affected the STAT3 signaling pathway by facilitating dephosphorylation of STAT3 via protein tyrosine phosphatase sigma (PTPRS) in hepatocellular carcinoma, a previously unknown phosphatase of STAT3 [22]. BMP10 belongs to the transforming growth factor (TGF) superfamily and participates in heart development [23]. BMP10 knockout results in lethal arteriovenous malformations in embryonic mice [24]. In contrast to other BMP members, BMP10 is exclusively expressed in the heart [25]. Moreover, BMP10 is involved in the postnatal maintenance of heart function. As one of the most potent endothelial activators of BMP-signaling, BMP10 attenuates endothelial apoptosis via ALK1 [26]. It is elevated in adult cardiomyocytes in response to pressure load-induced cardiac hypertrophy [27]. It regulates cardiac growth, cardiac apoptosis, and embryonic survival [28]. Therefore, it is possible that BMP10 protects against DOX-induced cardiac injury.

Based on the literature described above, we hypothesized that BMP10 has a cardioprotective effect in DOX-induced cardiac injury that is dependent on STAT3 activation. Our study aimed to explore the role of BMP10 in DOX-induced cardiac injury and confirm the mediation of STAT3. It could suggest a potential treatment for DOX-induced cardiac injury caused by DOX in clinical medicine.

## Materials and methods

### Reagents

DOX (purity ≥98%) was purchased from Sigma-Aldrich (St. Louis, MO, USA). Antibodies for glyceraldehyde-3-phosphate dehydrogenase (GAPDH), B-cell lymphoma-2 (BCL-2), bcl2-associated X protein (BAX), phosphorylated STAT3 (P-STAT3), and total STAT3 (T-STAT3) were purchased from Cell Signaling Technology (Danvers, USA). Antibodies against BMP10 were obtained from R&D Systems (Minneapolis, MN, USA). All antibodies were diluted 1000 fold for use. Recombinant human BMP10 was obtained from Abcam (Cambridge, UK). The adeno‐associated virus 9 (AAV9) was purchased from DesignGene Biotechnology (Shanghai, China). The ApopTag® Plus In Situ Apoptosis Fluorescein Detection Kit (Millipore, Billerica, USA) was used for TdT-mediated dUTP nick-end labeling (TUNEL) staining. Dihydroethidium (DHE) was obtained from Nanjing KeyGen (Jiangsu, China). The ELISA kits for NADPH oxidase, 4-HNE, SOD, and CAT were purchased from Nanjing Jiancheng Bioengineering Institute (Nanjing, China).

### Animals

All study procedures were approved by the Animal Care and Use Committee of the Renmin Hospital of Wuhan University. C57/BL6 mice (male, 8 weeks old) were acquired from the Institute of Laboratory Animal Science (Beijing, China). A pathogen‐free barrier system under controlled temperature (20–25°C) and humidity (50 ± 5%), was provided to the mice. To specifically overexpress or silence BMP10 in the myocardium, mice were intravenously injected with AAV9 carrying *bmp10* or sh*bmp10* at a dose of 1 × 10^11^ viral genome particles/mouse via tail vein [29]. To induce cardiac injury, DOX was intraperitoneally injected at a single dose of 15 mg/kg four weeks later [30]. Myocardial damage markers in the serum were detected on the 3rd day after DOX injection. To test the hypothesis that BMP10 cardioprotection depends on STAT3 activation, STAT3 conditional floxed mice were generated as described previously [31] and obtained from Cyagen (Suzhou, China). The Jackson Laboratory provided α-Mhc-Cre mice. STAT3 conditional floxed mice were mated with mice carrying α-Mhc-Cre, resulting in STAT3-cKO mice. Tamoxifen was administered to STAT3-cKO mice to induce STAT3-deficiency, as described previously [32].

Four weeks after DOX injection, the mice were sacrificed following hemodynamics and echocardiography. The mice were dissected, their hearts were removed, and the heart weight (HW) and tibial length (TL) were measured. Heart tissues were placed in 10% KCl to arrest the heart at the end of diastole, fixed in 10% formalin, embedded in paraffin, and cut into slices. To investigate whether exogenous BMP10 supplementation reversed DOX-induced advanced cardiac dysfunction, mice were randomly treated with recombinant human BMP10 (rhBMP10) or saline by intraperitoneal injection for seven days starting two weeks after DOX exposure. Cardiac function was evaluated by echocardiography.

### Cell culture

Cardiac myocytes (CMs) were isolated from neonatal rats as described in our previous studies [33, 34]. CMs were cultured in Dulbecco’s modified Eagle’s Medium/ F-12 (Gibco, NY, USA) supplemented with 10% fetal bovine serum (Gibco). The cells were then incubated in a humidified incubator at 37 C with 5% CO2. Acclimatization culture was performed for 48 h prior to the trial. To silence STAT3, CMs were pre-infected with si*stat3* (50 nmol/L) using Lipofectamine 6000 for 4 h. For BMP10 overexpression, cells were transfected with adenovirus, diluted at 100 MOI, for 4 h as reported in our previous studies [34].

### Echocardiography and hemodynamic evaluation

According to our previous experience [34, 35], mice were anesthetized with 1.5% isoflurane, and M-mode images were recorded using a MyLab 30CV ultrasound system (Genoa, Italy). Ejection fraction (EF) and fractional shortening (FS) data were obtained from the horizontal short-axis section of the left ventricular papillary muscle. Hemodynamic evaluation was performed using a PowerLab system (Oxford, UK). A microtip catheter transducer (Houston, USA) was embedded into the left ventricle to measure hemodynamics.

### Western blot and quantitative real‐time PCR

Based on some previous reports [34, 36], RIPA reagents (Invitrogen) were used to obtain total protein from the murine heart tissue. The protein concentrations were normalized to a consistent level. The proteins were separated by sodium dodecyl sulfate-polyacrylamide gel electrophoresis (SDS-PAGE) and then transferred to polyvinylidene fluoride membranes (Millipore). Subsequently, nonfat milk was used to block the membrane for 30 min to avoid interference from impurities. Antibodies matching the target protein were applied next, and incubated overnight in a 4°C refrigerator to bind the target protein. After incubation with secondary antibodies for 40 min, proteins were visualized using enhanced chemiluminescence (ECL) reagents and scanned using Bio‐Rad ChemiDoc XRS. For data analysis, the protein expression levels were normalized to those of reference proteins.

A TRIzol kit was used to isolate and obtain total ribonucleic acid (RNA) from murine hearts. Reverse transcription was performed using a Transcriptor First Strand cDNA Synthesis Kit (Roche). For comparison of relative mRNA expression, polymerase chain reaction was performed using a LightCycler 480 SYBR Green Master Mix (Roche). For data analysis, the mRNA expression was normalized to that of GAPDH.

### ROS detection and Apoptosis

Dihydroethidium (DHE) was used to determine the ROS levels in murine heart, as described in our previous study [37]. Sections of murine heart were incubated with DHE in the absence of light at 37 C for 30 min. After visualizing the nucleus with DAPI, the sections were photographed using an Olympus IX53 fluorescence microscope. To further assess the oxidative stress levels, we measured nicotinamide adenine dinucleotide phosphate (NADPH) oxidase activity, 4-hydroxynonenal (4-HNE) levels, superoxide dismutase (SOD) activity, and catalase (CAT) activity in the heart using commercially available kits [38]. Apoptosis was assessed by TUNEL staining [35]. The images were captured by a special OLYMPUS DX51 fluorescence microscope (Tokyo, Japan). All data were analyzed using a digital analysis software (Image-Pro Plus 6.0, Media Cybernetics, Bethesda, USA). All the slides were examined by two authors in a blinded manner. The positive expression was counted randomly from 20 fields in each section, and counted at least three sections from each heart. The numbers of TUNEL+ cells were calculated as percentage of total TUNEL+cells counted. Commercial kits detected Caspase-3 activity and cytochrome C (Cyt-C) levels simultaneously.

### Biochemical analysis

On the 3rd day after DOX treatment, blood was collected performed through the orbital vein of the mice. An automatic biochemical analyzer (ADVIA® 2400; Siemens) was used to determine the levels of creatine kinase isoenzymes (CK-MB), lactate dehydrogenase (LDH), and cardiac isoform of troponin T (cTnT) [35].

### Statistical analysis

All data were analyzed using SPSS22.0 software. Data are presented as the mean ± standard error of the mean. Statistical significance between groups was examined using the Student’s t-test. One‐way analysis of variance (ANOVA) was performed to compare the data derived from multiple groups. Statistical significance was set at *p* < 0.05.

## Results

Use of DOX is limited in antitumor treatment due to its severe cardiotoxicity. Cardiac oxidative stress and apoptosis are the main characteristics of DOX-induced cardiac injury. As a cardioprotective factor, BMP10 activates STAT3 in cardiomyocytes and protects cardiac function. Our study aimed to explore the role and mechanism of action of BMP10 in DOX-induced cardiac injury. We hypothesized that BMP10 is beneficial in DOX-induced cardiac injury, and its function depends on STAT3 activation; the obtained results confirmed our hypotheses.

### BMP10 alleviated DOX-induced cardiac injury

**Figure 1.**
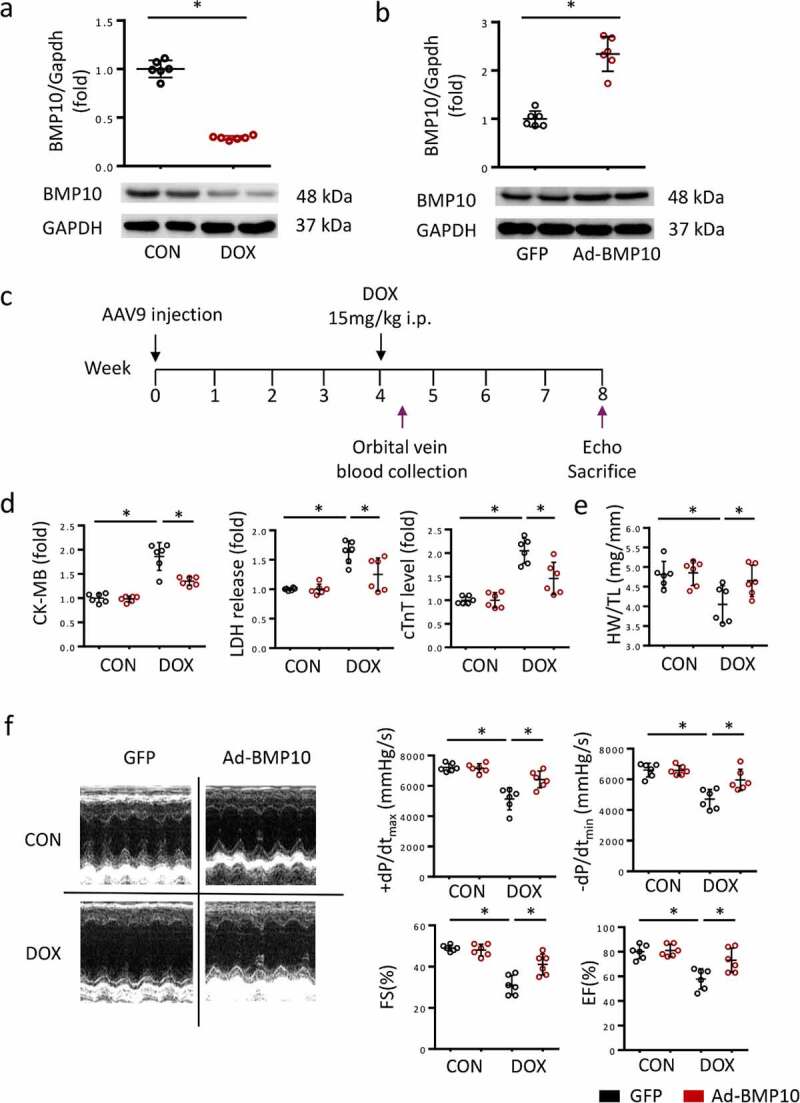
BMP10 overexpression alleviates DOX-induced cardiac injury. **a** BMP10 expression in mice hearts was determined by Western blotting 5 days after DOX injection (n = 6). **b** Cardiac BMP10 protein expression in mice with or without BMP10 overexpression (n = 6). **c** Schematic protocol for AAV9 and DOX treatment. **d** Biochemical determination of CK-MB, LDH, cTnT serum levels (n = 6). **e** Measurement results of mouse heart weight/tibial length (HW/TL) (n = 6). **f** Fractional shortening (FS), ejection fraction (EF) and ±dp/dt determined by echocardiography and hemodynamics (n = 6). *p < 0.05 vs corresponding group.

BMP10 expresses abundantly in the heart, which was consistent with our western blot results. However, BMP10 protein levels were significantly reduced in DOX-treated hearts (Figure 1(a)), suggesting that BMP10 may be involved in DOX-induced cardiotoxicity. To explore the role of BMP10, the AAV9 system was used to overexpress cardiac-specific BMP10 (Figure 1(c)). As shown in Figure 1(b), cardiac BMP10 protein levels were significantly higher in mice infected with AAV9-AdBMP10 than in controls infected with AAV9-green fluorescent protein. To evaluate the cardiac injury caused by DOX, we collected blood from the orbital vein of mice on the third day after DOX treatment. Consistent with previous results, DOX treatment significantly elevated serum CK-MB, LDH, and cTnT levels, thereby predicting severe cardiac injury. However, we found that mice with cardiac-specific overexpression of BMP10 had reduced levels of elevated CK-MB, LDH, and cTnT, indicating that BMP10 overexpression protects the heart from damage (Figure 1d). Moreover, the HW/TL ratio was reduced after DOX injection, and the phenomenon was significantly attenuated by BMP10 overexpression (Figure 1(e)). Consistent with the decreased levels of myocardial injury biomarkers, BMP10 overexpression alleviated DOX-induced cardiac dysfunction, as indicated by the preserved EF, FS, systolic function (assessed by +dp/dt), and diastolic function (as assessed by −dp/dt) (Figure 1(f)). Based on the above data, we suggested that BMP10 protects against DOX-induced cardiac injury and functional deterioration.

### BMP10 suppressed DOX-induced oxidative stress injury and myocardial apoptosis

**Figure 2.**
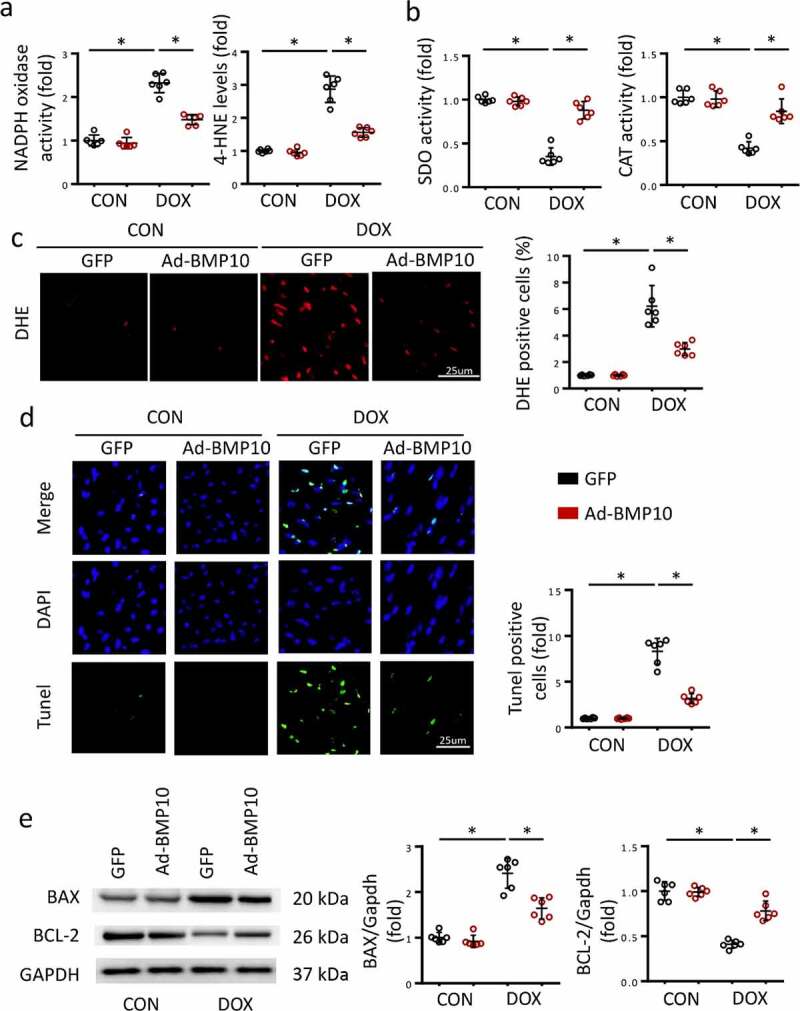
BMP10 overexpression protects the heart from oxidative stress injury and cardiac apoptosis in response to DOX. **a-b** Quantitative results of myocardial NADPH oxidase activity, 4-HNE level, SOD activity, and CAT activity (n = 6). **c** Representative DHE staining images and the statistical results (n = 6). **d** TUNEL staining and quantitative results in DOX-treated hearts with or without BMP10 overexpression (n = 6). **e** Western blotting and the quantitative results of apoptosis-related proteins (n = 6). *p < 0.05 versus the matched group.

Oxidative stress injury is a major feature of DOX-induced cardiac injury. To assess cardiac oxidative stress levels, we measured NADPH oxidase activity, 4-HNE levels, SOD activity, and CAT activity in the heart using commercially available ELISA kits. As expected, NADPH oxidase activity and LDH levels significantly increased after DOX treatment (Figure 2(a)), whereas SOD and CAT activities decreased (Figure 2(b)), indicating that DOX disrupts normal cardiac redox homeostasis. However, BMP10 overexpression partially reversed this adverse transformation (Figure 2(a–b)). DHE staining was performed to detect ROS in the heart. Consistent with our expectations, BMP10 overexpression sufficiently reduced the cardiac ROS overproduction caused by DOX (Figure 2(c)).

Massive cardiac apoptosis, caused by DOX-induced cardiac toxicity and ROS accumulation, contributes to the progression of DOX-induced cardiac injury. Given that BMP10 overexpression reduces ROS production, it would be reasonable to hypothesize that BMP10 suppresses cardiac apoptosis. To prove our hypothesis, TUNEL staining was performed to visualize apoptotic cells. Results showed DOX treatment to cause massive apoptosis, which was significantly inhibited by cardiac-specific BMP10 overexpression (Figure 2(d)). The anti-apoptotic protein Bcl-2 and cell death promoter BAX were detected in the hearts of mice after DOX injection. Suppression of cardiac apoptosis was further confirmed by molecular alterations, and western blotting showed BMP10 overexpression to reduce Bax expression and upregulated Bcl-2 levels (Figure 2(e)).

### BMP10 deficiency aggravated DOX-induced cardiac dysfunction, cardiac oxidative stress, and apoptosis

**Figure 3.**
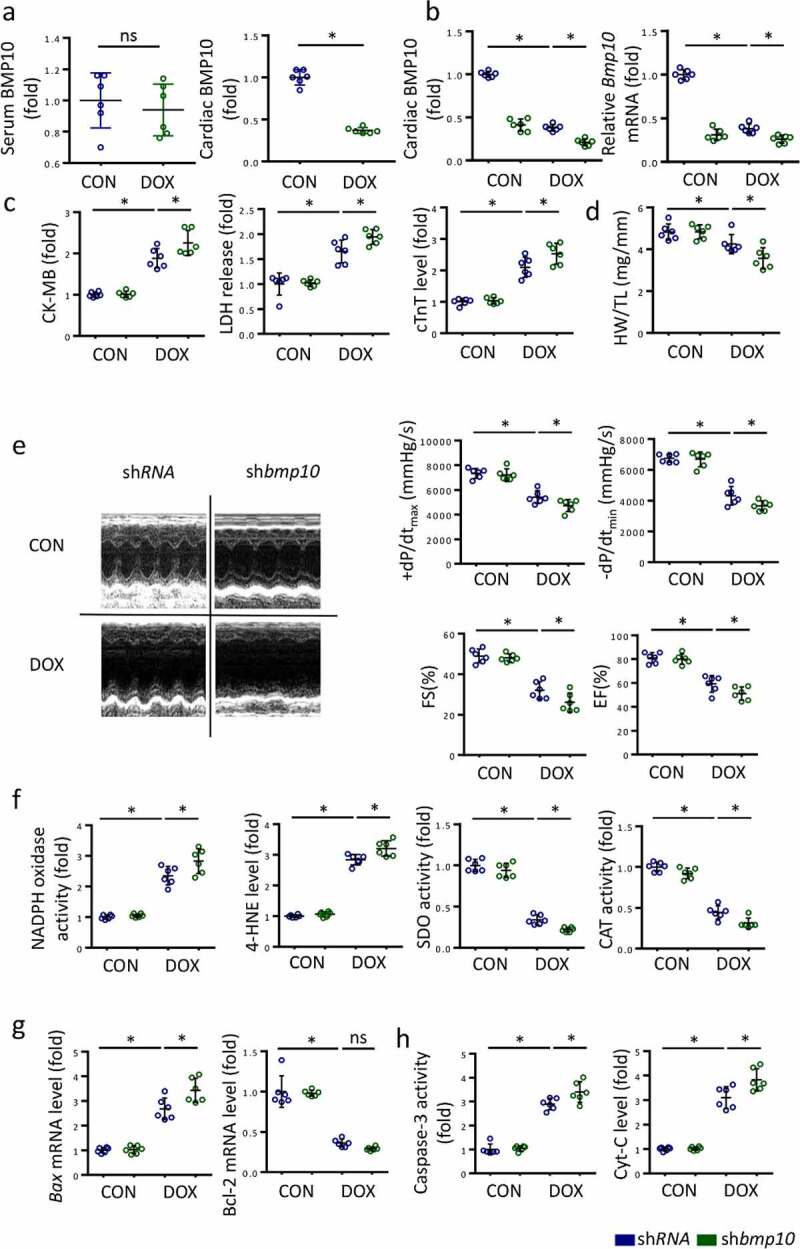
BMP10 deficiency worsens DOX-induced cardiac oxidative stress, apoptosis, and cardiac dysfunction. **a** Expression of BMP10 in serum and heart after DOX exposure (n = 6). **b** BMP10 levels in DOX-treated hearts with or without BMP10 silencing were detected by ELISA and qPCR (n = 6). **c** Biochemical determination of CK-MB, LDH, cTnT serum levels after three days of DOX treatment (n = 6). **d** Heart weight to tibial length ratio (HW/TL) (n = 6). **e** Cardiac function and hemodynamic parameters of mice (n = 6). **f** ELISA results and statistics of NADPH oxidase activity, 4-HNE level, SOD activity, and CAT activity (n = 6). **g** The mRNA levels of apoptosis-related genes Bax and BCL-2 were detected by qPCR (n = 6). **h** Quantitative results of myocardial Caspase-3 activity and Cyt-C level (n = 6). *p < 0.05 versus the matched group. NS means no significance.

To identify the source of BMP10 in response to DOX injury, we examined BMP10 in the serum and in heart using ELISA. We found that the level of BMP10 in serum did not significantly change after DOX treatment, whereas cardiac BMP10 levels were remarkably decreased in response to DOX stimulation (Figure 3(a)); this indicated that decrease in BMP10 mainly occurred in the myocardial tissue, rather than in blood circulation, during DOX stimulation. Therefore, we silenced BMP10 specifically in the myocardium using AAV9 carrying sh*bmp10* while myocardial injection of AAV9 carrying scrambled shRNA was used as a control. As shown in Fig. S1, we characterized the distribution of GFP expression in the murine heart following AAV9-mediated gene delivery by systemic injection and discovered that AAV9 system carrying sh*bmp*10 to specifically target cardiomyocytes,and protein levels of BMP10 was confirmed by western blot. The efficiency of Ad-sh*bmp10* was confirmed using ELISA and qPCR examination (Figure 3(b)). Three days after the intraperitoneal injection of DOX, we observed higher CK-MB, LDH, and cTnT levels in the serum of mice with cardiac-specific BMP10 silencing compared to that in the control (Figure 3(c)), indicating that BMP10 deficiency aggravated DOX-induced cardiac injury. Moreover, BMP10-deficient mice had lower HW/TL ratio than the DOX-treated control mice (Figure 3(d)). Furthermore, BMP10 deficiency worsened DOX-induced cardiac dysfunction, as reflected by EF, FS, and ±dp/dt in DOX-treated mice (Figure 3(e)). The results together implied that BMP10 deficiency aggravates DOX-induced cardiotoxicity in mice.

To investigate the effects of BMP10 deficiency on DOX-induced redox disorder and apoptotic cells, we identified the molecular changes representing the redox system and apoptosis. In DOX-exposed mice, cardiac-specific BMP10 silencing increased the pro-oxidant NADPH oxidase activity and 4-HNE levels, while decreasing antioxidant SOD activity and CAT activity (Figure 3(f)). Real-time PCR results demonstrated higher *Bax* mRNA levels in BMP10-deficient mice than in sh*RNA-*treated mice following DOX stimulation (Figure 3(g)). Unexpectedly, we found BMP10 deficiency to not alter *Bcl-2* mRNA levels in presence of DOX (Figure 3(g)). ELISA results also indicated that BMP10 deficiency aggravated apoptosis, as reflected by the higher levels of Capases-3 activity and Cyt-C (Figure 3(h)). Therefore, we concluded that BMP10 deficiency deteriorates DOX-induced cardiac oxidative stress and apoptosis.

### Stat3 deletion blocked the cardioprotective effect of BMP10 in mice with DOX treatment

**Figure 4.**
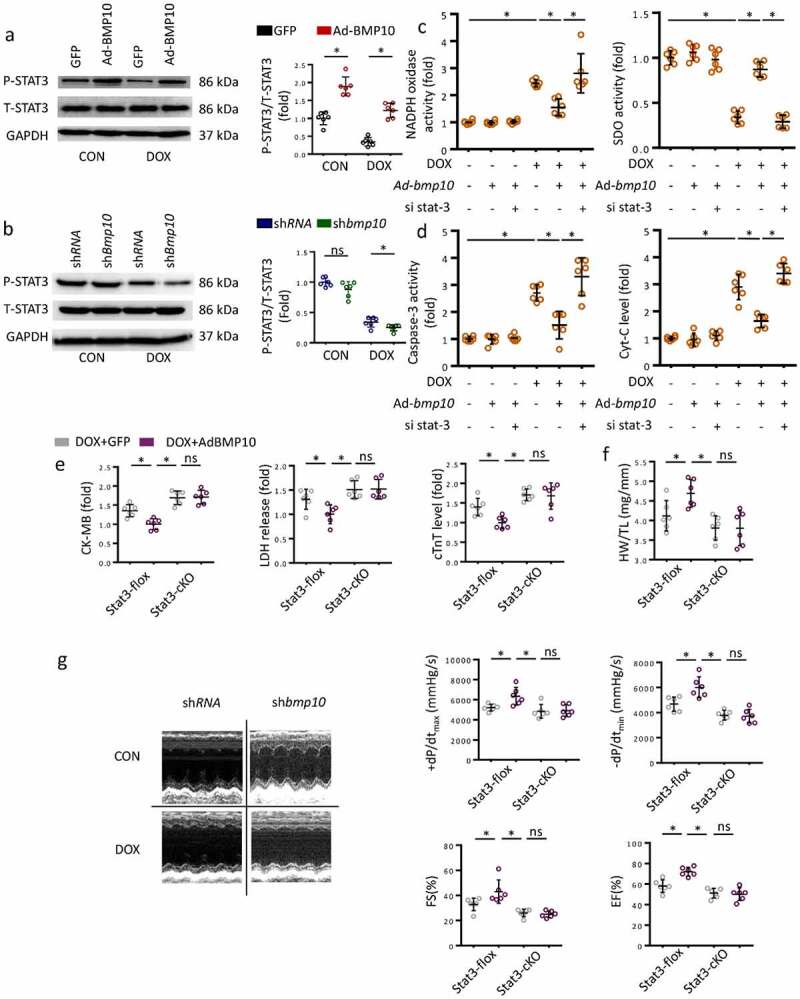
STAT3 is necessary for BMP10 to attenuate oxidative stress and apoptosis. **a** Western blot and analysis of STAT3 phosphorylation level in BMP10 overexpressed hearts (n = 6). **b** Western blot results and statistics of STAT3 phosphorylation in DOX-treated hearts with or without BMP10 silencing (n = 6). **c** Results of NADPH oxidase and SOD activities related to oxidative stress in cardiomyocytes. **d** ELISA results of Caspase3 activity and Cyt-C level in cardiomyocytes (n = 6). **e** Biochemical determination of CK-MB, LDH, cTnT serum levels in STAT3-flox and STAT3-cKO mice with or without BMP10 overexpression after DOX-treated (n = 6). **f** Measurement results of HW/TL in STAT3-flox and STAT3-cKO mice (n = 6). **g** Echocardiography and hemodynamic statistics after 5 days of DOX treatment in STAT3-flox and STAT3-cKO mice (n = 6). *p < 0.05 versus the matched group. NS means no significance.

STAT3 is considered a key signal responsible for cellular oxidative stress and apoptosis. Previous studies had shown the activation of STAT3 signaling to attenuate cardiac dysfunction in DOX-exposed mice. Here, the p-STAT3 level was observed to be decreased significantly in response to DOX cardiotoxicity in vivo (Figure 4(a-b)). However, myocardial overexpression of BMP10 substantially activated STAT3 phosphorylation, both in the control and in the DOX-treated mice (Figure 4(a)). BMP10 silencing resulted in lower p-STAT3 levels upon DOX treatment, whereas there was no difference in p-STAT3 levels in saline-treated mice (Figure 4(b)). To gain evidence that the protective effects of BMP10 were mediated by STAT3 activation, we treated the cells with si*stat3*. We found that STAT3 silencing abolished the effect of BMP10 overexpression on redox homeostasis maintenance, as reflected by the decrease in NADPH activity being reversed, and the increase in SDO activity being counteracted (Figure 4(c)). In addition, the anti-apoptotic effect of BMP10 overexpression was abolished by si*stat3* incubation, as indicated by the results of Caspase-3 activity and Cyt-C levels (Figure 4(d)). Given that STAT3 is linked to inflammation-associated tumorigenesis [39], we explored whether inflammatory mediators could be involved in the cardioprotective effects of BMP10. BMP10 was found to decrease *TNF-α* and *IL-1β* mRNA levels in DOX-treated NRCMs. ROS scavenging by N-acetyl-L-cysteine (NAC) inhibited DOX-induced inflammation. Unexpectedly, pretreatment of BMP10-overexpressing cells with NAC showed no further attenuation of DOX-induced transcription of inflammatory factors (Fig. S2). This finding implied that BMP10 alleviated DOX-induced inflammation via ROS scavenging. Collectively, the data indicated that the antioxidant and anti-apoptotic effects of BMP10 are dependent on STAT3 activation.

We further explored whether STAT3 could be responsible for the cardioprotective role of BMP10 in DOX-treated mice. Cardiac-specific deletion of Stat3 (Stat3-cKO) was used to verify this hypothesis. Consistent with the above results, overexpression of BMP10 attenuated cardiotoxicity in Stat3-flox mice (Figure 4(e-g)). As shown in Figure 4(e), Stat3-cKO abolished the cardioprotective effects of BMP10 overexpression, as indicated by higher CK-MB, LDH, and cTnT levels even in BMP10 overexpressing mice. In addition, Stat3-cKO eliminated the improvement of HW/TL by BMP10 (Figure 4(f)). Furthermore, Stat3-cKO abrogated the improvements in EF, FS, and dp/dt with BMP10 overexpression during DOX administration (Figure 4(g)). To enhance the value for clinical applications, we examined whether BMP10 could attenuate DOX-related cardiotoxicity. Mice were randomly administered recombinant human BMP10 or vehicle treatment for one week beginning two weeks after DOX treatment. BMP10 treatment reversed the impaired cardiac function after DOX injection, as indicated by EF and FS values (Fig. S3).

## Discussion

Based on our current data, BMP10 expression decreased in DOX-induced cardiotoxicity in mice, and BMP10 overexpression alleviated the DOX-induced cardiac injury and improved cardiac function. Furthermore, BMP10 overexpression repaired the DOX-induced ROS accumulation and cardiac apoptosis, whereas BMP10 deficiency aggravated DOX-induced cardiac injury. Our results also demonstrated that BMP10 activated STAT3, decreasing the production of superoxide and subsequent cell apoptosis. Importantly, recombinant BMP10 could treat DOX-induced myocardial damage.

DOX is an effective broad-spectrum anti-tumor agent. Its antitumor mechanism involves interfering with double-stranded DNA repair, hindersing DNA replication and transcription, and eventually causing cancer cell death [6]. However, while killing tumor cells, DOX also damages cellular DNA, leading ROS overproduction and apoptosis [40]. Therefore, DOX is often accompanied by diverse and severe anticancer side effects [41]. In particular, DOX has been reported to aggregate in the heart, causing prominent and fatal cardiotoxicity [35]. DOX treatment leads to progressive cardiomyopathy and cardiac dysfunction, limiting its clinical application [42]. Numerous studies have demonstrated that redox disorders and ROS overproduction are vital features of DOX-induced cardiac injury [12, 34, 43]. Despite the massive number of studies focusing on cardiac injury caused by DOX, no effective therapeutic agent is available yet for clinical treatment. More mechanistic studies would be required in future to provide ideas for drug development for the reduction of cardiac damage caused by DOX application. In the present study, oxidative stress, apoptosis, cardiac injury, and cardiac dysfunction were alleviated by BMP10 overexpression in DOX-treated mice. BMP10 supplementation alleviated DOX-induced cardiac contractile dysfunction, which may provide a potential therapy for managing DOX-induced cardiac injury.

As a peptide growth factor, BMP10 belongs to the BMP family [44]. BMPs mediate various developmental events and physiological processes in insects and mammals [45, 46]. Although the protein structures are similar, each member of the BMP family has a unique amino acid sequence, different distribution characteristics, and diverse physiological functions [47]. Previous reports had shown that BMP2, BMP4, BMP5, BMP6, BMP7, and BMP10 are mainly expressed in the heart, BMP10 especially having a specific and rich cardiac distribution [48, 49]. BMP10 has been reported to be a cardioprotective factor that triggers intracellular signal transduction and regulates cardiac physiological and pathological processes [27, 50]. For example, BMP10 promotes cardiomyocyte survival and inhibits excessive deposition of extracellular matrix, thus preventing adverse cardiac remodeling [51]. However, the biological function of BMP10 in the heart has not been fully elucidated, and its mechanism of action is still unclear, especially in DOX-induced cardiac injury. Our data showed that the level of cardiac BMP10, not serum BMP10, was decreased in response to DOX exposure. Alteration of the BMP10 expression pattern implied its potential role in DOX-induced cardiotoxicity. BMP10 was then overexpressed in mouse heart to verify the same. We observed that BMP10 not only attenuated cardiac injury but also improved cardiac function in DOX-treated mice. The role of oxidative stress as a mediator of DOX toxicity in the heart had been reported in previous studies. Consistently, we found that DOX-treated mice had increased generation of ROS and 4-HNE by NADPH oxidase [52] and decreased levels of the major antioxidant enzymes SOD and CAT. Importantly, amplifying oxidative stress can lead to exacerbated apoptosis [53], which can, in turn, provoke ROS overproduction, creating a vicious cycle that further aggravates cardiac injury and dysfunction. Based on our results, we investigated whether BMP10 protects against DOX-induced cardiac injury via antioxidant and anti-apoptotic mechanisms. In our study, BMP10 overexpression remarkably alleviated oxidative damage and apoptosis in the hearts of DOX-treated mice. The data demonstrated that BMP10 plays a cardioprotective role in DOX-mediated cardiotoxicity via antioxidant and anti-apoptotic mechanisms.

The STAT protein family comprises of a widespread group of signaling proteins that can be activated in response to various cytokine stimuli [54]. Within the STAT family, STAT3 is thought to be involved in hematopoiesis, immunity, and cancer progression [^55–57^]. STAT3 is also implicated in cardiac conditioning. STAT3 activation alleviates cardiac fibrosis via macrophage polarization regulation [58] and STAT3 regulates mitochondrial function to mediate cardioprotection during ischemia-reperfusion injury [59]. Recently, impaired STAT3 signaling was reported to contribute to the etiology of DOX-induced cardiomyopathy [20]. Similar to previous reports [20], we observed a decline in p-STAT3 levels in DOX-treated murine hearts. STAT3 activation has been reported to up-regulate the levels of antioxidant enzymes and anti-apoptotic proteins. Here, we found that DOX decreased the levels of STAT3 phosphorylation, whereas BMP10 overexpression could enhance the same. STAT3 deficiency completely abolished the protective effect of BMP10, implying that BMP10 protected against DOX-induced cardiotoxicity by activating STAT3. Further investigation indicated that BMP10 in fact reversed the pre-established cardiac injury caused by DOX. These findings collectively suggested that BMP10 supplementation could be a potential treatment for DOX-induced cardiomyopathy. We identified non-canonical STAT3 activation as a crucial mediator of the BMP10-induced antioxidant and anti-apoptotic effects in DOX-induced cardiac injury. Given the potential oncogenic risk of STAT3 agonists [60], cardiac specific overexpression of BMP10 may be safer than the use of STAT3 agonists. However, We did not use CM markers in TUNEL and DHE staining, and used whole heart lysates for western blot. In addition, Most of the data are observed in vivo study. To further clarify the role of BMP10 on cell type, additional studies are needed to define more clearly in vitro.

## Conclusion

In summary, our study confirmed that BMP10 plays a vital role in DOX-induced cardiac injury. We found BMP10 to alleviate DOX-induced cardiac oxidative stress and apoptosis, and improve cardiac function. Mechanistically, we demonstrated the cardioprotective effect of BMP10 to depend on STAT3 activation. Thus, BMP10 administration could be a potential approach to alleviate DOX-induced cardiac injury in patients with cancer.

## Supplementary Material

Supplemental Material

## Data Availability

Data supporting the findings of this study could be obtained from the corresponding author upon reasonable request.
