# Supplementary material for: Bone morphogenetic protein 10 alleviates doxorubicin-induced cardiac injury via signal transducer and activator of transcription 3 signaling pathway
Source: Bioengineered. 2022 Mar 16;13(3):7471–84. doi: 10.1080/21655979.2022.2048994 (PMC9208532; doi:10.1080/21655979.2022.2048994)
Supplement: Supplemental Material [file KBIE_A_2048994_SM1607.pdf]

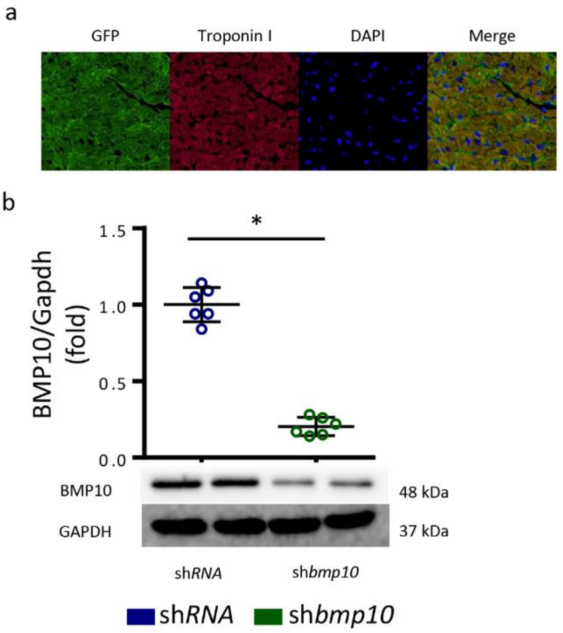

Figures S1

Infection and expression of AAV9-GFP-shBMP10 in mice hearts. (A). Verification of cellular uptake of GFP-labeled AAV9-shBMP10 three days after infection. Cardiomyocytes marker Troponin I. Scale bar = 100μm. (B) The BMP10 protein levels in hearts at 4 weeks after adenovirus infection (n = 6). *p<0.05 versus the Ad-shRNA.

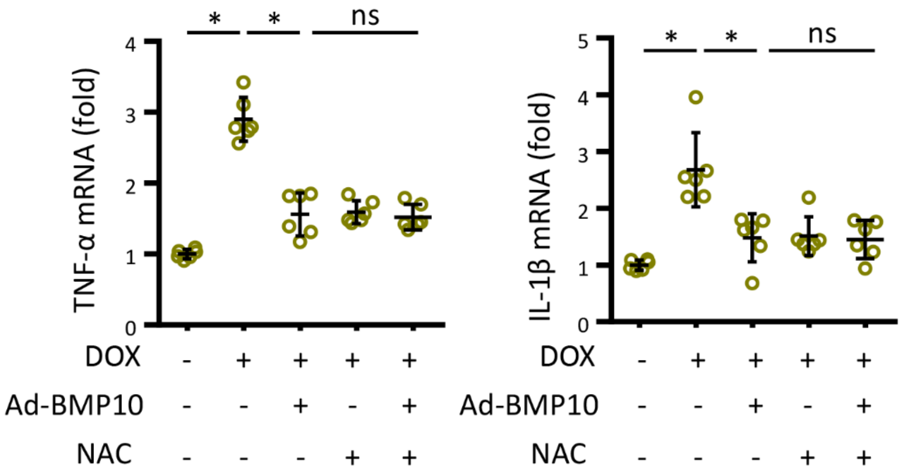

Figure S2

The mRNA levels of TNF-α and IL-1β in cardiomyocytes were detected by qPCR. *$p < 0.05$ versus the matched group. NS means no significance.

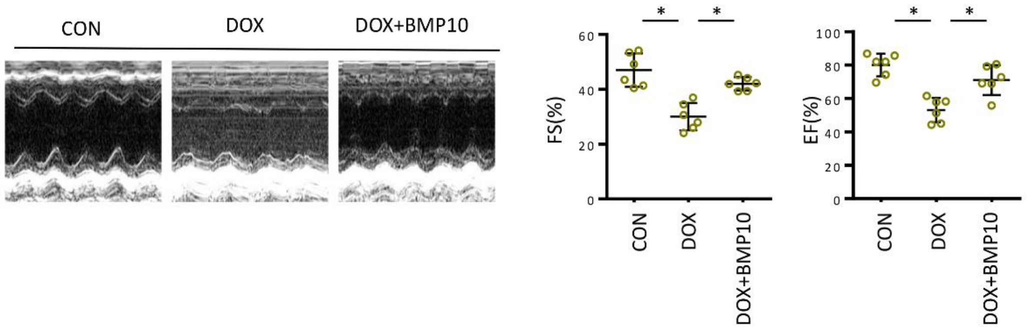

Figure S3
Cardiac function in DOX-treated mice with or without exogenous BMP10 supplementation (n = 6).
*p< 0.05 versus the matched group.
